# Intrinsic Capacity and Its Relationship With Life Space Area (INCREASE) in Community-Dwelling Older Adults

**DOI:** 10.1093/geroni/igab046.1406

**Published:** 2021-12-17

**Authors:** Jia Qi Lee, Yew Yoong Ding, Laura Tay, Aisyah Latib, Yee Sien Ng

**Affiliations:** 1 Duke-NUS Graduate Medical School, Singapore, Singapore; 2 Geriatric Education and Research Institute, Singapore, Singapore; 3 Sengkang General Hospital, Singapore, Singapore; 4 Singapore General Hospital, Singapore, Singapore

## Abstract

Intrinsic capacity (IC), defined as ‘the composite of all physical and mental capacities of an individual’, is of increasing interest in geriatrics as a potential multidimensional measure of health in older adults. According to the International Classification of Functioning, Disability and Health (ICF) framework, IC, through its interactions with environmental factors, determines a person’s participation in the community. However, there is lack of empirical evidence demonstrating this association. The primary aim of this study was to examine the association of IC with Life Space Area (LSA; a measure of participation) among community-dwelling older adults. The secondary aim was to determine whether age and gender modify this relationship. Cross sectional analysis was performed on data from the Individual Physical Proficiency Test for Seniors (IPPT-S) study conducted in the Northeastern region of Singapore. Standardized IC factor scores were calculated through confirmatory factor analysis using variables that represented the 5 IC domains. Association of IC with LSA and its effect modification by age and gender were examined with regression analyses. The study included 751 participants with mean age of 67.6 and mean LSA score of 88.6. IC showed a positive and significant association with LSA (B=6.33, P<0.001) and the effect remained significant even after controlling for potential confounders (B=4.76, P<0.001). Age and gender did not show significant modification on this relationship. Our findings support the empirical rigour of the ICF framework and provide guidance for healthcare providers who aim to enhance life space mobility and promote healthy aging in older adults.

